# Immunomodulatory Effect and Bone Homeostasis Regulation in Osteoblasts Differentiated from hADMSCs via the PD-1/PD-L1 Axis

**DOI:** 10.3390/cells11193152

**Published:** 2022-10-07

**Authors:** Seung-Cheol Lee, Min Kyoung Shin, Bo-Young Jang, Seung-Ho Lee, Min Kim, Jung-Suk Sung

**Affiliations:** Department of Life Science, Dongguk University-Seoul, Goyang 10326, Korea

**Keywords:** PD-1/PD-L1 axis, osteoblasts, bone regeneration, bone homeostasis, hADMSCs

## Abstract

Human mesenchymal stem cells (hMSCs) are promising candidates for stem cell therapy and are known to secrete programmed death-1 (PD-1) ligand 1 (PD-L1) regulating T cell-mediated immunosuppression. Given the limitations of current stem cell therapy approaches, improvements in immunomodulatory capacity and stem cell differentiation efficacy are needed. In this study, we propose novel strategies to overcome the challenges that remain in hMSC-mediated bone regeneration. We found that PD-1 is highly expressed in osteoblasts, and the PD-1/PD-L1 axis mediated the decreased proinflammatory cytokine expressions in differentiated osteoblasts cocultured with human adipose derived mesenchymal stem cells (hADMSCs). Moreover, the decrease was attenuated by PD-1/PD-L1 pathway inhibition. Osteogenic properties including osteogenic gene expression and calcium deposits were increased in osteoblasts cocultured with hADMSCs compared with those that were monocultured. Osteoblasts treated with PD-L1 and exosomes from hADMSCs also exhibited enhanced osteogenic properties, including calcium deposits and osteogenic gene expression. In our cocultured system that mimics the physiological conditions of the bone matrix, the PD-1/PD-L1 axis mediated the increased expression of osteogenic genes, thereby enhancing the osteogenic properties, while the calcium deposits of osteoblasts were maintained. Our results provide the therapeutic potentials and novel roles of the PD-1/PD-L1 axis in bone matrix for modulating the bone properties and immunosuppressive potentials that can aid in the prevention of bone diseases via maintaining bone homeostasis.

## 1. Introduction

Human mesenchymal stem cells (hMSCs) are known to possess great potential in stem cell therapy due to their immunosuppressive effects and multipotency that enables them to differentiate into diverse cell types, including osteoblasts, adipocytes, chondrocytes, and neuronal cells [1,2]. hMSCs migrate into injured tissue and repair the damaged sites in response to specific homing signals [3]. They possess various types of cell surface receptors and the immunosuppressive properties of hMSCs are known to be mediated via interaction between ligands and their surface receptors [4]. Likewise, we previously demonstrated the regulatory mechanism and the function of CXCR6 during the differentiation process of hMSCs [5,6]. The receptors on hMSCs and differentiated cells play various biological roles in, for example, cell homing, macrophage polarization, and the acceleration of differentiation [1,5,7].

hMSCs also secrete various biological factors, including chemokines, extracellular vesicles, and growth factors that are related to intracellular communication [8]. Programmed death-1 (PD-1) ligand 1 (PD-L1) is one of the hMSC-secreted agents that contribute to T cell-mediated immunosuppression under physiological conditions [9]. PD-L1 expressed by tumor cells is known to interact with its receptor PD-1 in T cells and inhibits T cell activation, proliferation, and survival, eventually leading to the progression of tumors [10]. However, a better understanding of the immunosuppressive role of the PD-1/PD-L1 axis may provide us with insights that will be especially beneficial for those fields that deal with immunomodulatory functions [11]. Recent studies have revealed that PD-L1 exists in extracellular form and that exosomal PD-L1 is mostly secreted by tumor cells, including melanoma cells, breast cancer cells, and colon cancer cells, modulating immune responses [12,13]. hMSCs are also known to secrete exosomal PD-L1, but what we know about the PD-1/PD-L1 axis in stem cell research is limited to its immunosuppressive functions [14].

hMSCs can be isolated from various tissue and organs such as bone marrow, adipose tissue, and umbilical cord [15]. Among them, the adipose-tissue-derived mesenchymal stem cells possess great potential for bone tissue engineering due to the easy isolation processes, significative quantities in harvesting, and high paracrine activity through the secretion of various bioactive molecules [16].

Bone is a complex and dynamic organ that is constantly being remodeled, and bone metabolism is balanced through the activity of osteoblasts and osteoclasts in tissues [17,18]. Osteoblasts are differentiated from pluripotential mesenchymal cell lineages, whereas osteoclasts arise from immune cells such as monocytes–macrophages lineages [19]. It is well known that the PD-1/PD-L1 axis plays a role in osteoclast formation and activity, reflected by the fact that osteoclasts are differentiated from immune cells [20,21]. However, the role of the PD-1/PD-L1 axis in osteoblasts and its modulatory function on the bone matrix is yet to be studied.

Here, we first demonstrate the regulatory function and immunomodulatory effects of the PD-1/PD-L1 axis on osteogenic properties in osteoblasts as well as its regenerative, homeostasis-maintaining effects in a cocultured system of osteoblasts and osteoclasts.

## 2. Materials and Methods

### 2.1. hADMSCs Culture and Adipogenic or Osteogenic Differentiation

Human adipose-derived mesenchymal stem cells (hADMSCs) were purchased from CEFO (Seoul, Korea). The cells with biological passage number 4 were seeded into appropriate cell culture dishes at the density of 6000 cells/cm^2^ and cultured in hADMSC growth medium (CEFO, Seoul, Korea) until cells were 80% confluent. Adipogenic differentiation was induced for 21 days in high-glucose Dulbecco’s Modified Eagle Medium (DMEM) (Gibco, Amarillo, TX, USA), containing 1% penicillin–streptomycin (Gibco, Amarillo, TX, USA), 10% fetal bovine serum (Alphabioregen, Boston, MA, USA), 1 μM dexamethasone (Sigma-Aldrich Chemicals, St. Louis, MO, USA), 100 μM indomethacin (Sigma-Aldrich Chemicals, St. Louis, MO, USA), 10 μg/mL insulin (Welgene, Daegu, Korea), and 500 μM 3-isobutyl-1-methylxanthine (Sigma-Aldrich Chemicals, St. Louis, MO, USA). Osteogenic differentiation was induced by culturing cells for 21 days in osteogenic differentiation medium containing low-glucose DMEM (Gibco, Amarillo, TX, USA), 1% penicillin–streptomycin (Gibco, Amarillo, TX, USA), 10% fetal bovine serum (Alphabioregen, Boston, MA, USA), 0.1 μM dexamethasone (Sigma-Aldrich Chemicals, St. Louis, MO, USA), 10 mM β-glycerophosphate (Sigma-Aldrich Chemicals, St. Louis, MO, USA), and 50 μM L-ascorbic acid-2-phosphate (Sigma-Aldrich Chemicals, St. Louis, MO, USA).

### 2.2. Oil Red O (ORO) Staining

Adipocytes were washed twice with PBS and fixed with 10% formalin (Sigma-Aldrich Chemicals, St. Louis, MO, USA) for 10 min. After fixation, cells were washed with PBS and 60% isopropanol (Sigma-Aldrich Chemicals, St. Louis, MO, USA) and dried. ORO solution (Sigma-Aldrich Chemicals, St. Louis, MO, USA) was used to stain the cells at room temperature for 30 min. Stained cells were washed twice with distilled water and imaged by microscopy (Leica, Wetzlar, Germany). To quantify the remaining ORO-stained cells, the dye was dissolved with 100% isopropanol and absorbance was measured at 492 nm with a microplate reader (Tecan, Männedorf, Switzerland).

### 2.3. Alizarin Red S (ARS) Staining

Osteoblasts were washed twice with PBS (Biosolution, Seoul, Korea) and fixed with 10% formalin (Sigma-Aldrich Chemicals, St. Louis, MO, USA) for 15 min. After fixation, cells were washed with PBS (Biosolution, Seoul, Korea) and then treated with ARS staining solution (Sigma-Aldrich Chemicals, St. Louis, MO, USA) for 45 min at room temperature in the dark. Next, cells were washed five times with distilled water and imaged by microscopy (Leica, Wetzlar, Germany). To quantify the ARS-stained cells, they were dissolved with 10% cetylpyridinium chloride (Sigma-Aldrich Chemicals, St. Louis, MO, USA) and absorbance was measured at 570 nm with a microplate reader (Tecan, Männedorf, Switzerland).

### 2.4. RNA Extraction and Real-Time PCR

Total mRNA from cells was isolated using RNeasy kits (Quiagen, Hilden, Germany) according to the manufacturer’s protocol. cDNA was synthesized with 0.5 µg of total mRNA template using Reverse Transcription Master Premix (ELPIS, Daejeon, Korea) according to the manufacturer’s instructions. To amplify cDNA, 40 cycles of PCR were performed with SYBR Green PCR Master Mix (KAPA Biosystem, Wilmington, NC, USA). The expression level of each target gene was normalized with an internal control, GAPDH. The specific oligonucleotide primers used in this study are listed in Table 1.

### 2.5. Immunofluorescence Staining

Cells were fixed with 4% paraformaldehyde (Sigma-Aldrich Chemicals, St. Louis, MO, USA) for 10 min, then washed twice with ice cold TBST, and 1% bovine serum albumin was used to block the samples for 30 min. Cells were then incubated with human PD-1 (R&D Systems, Minneapolis, MN, USA) primary antibody at 4 °C overnight. Donkey anti-goat IgG NorthernLights (R&D Systems, Minneapolis, MN, USA) was added as secondary antibody for 1 h. Nuclei were counterstained with 4,6-diamidino-2-phenylindole (DAPI). Confocal fluorescent images were obtained with a confocal microscope (Carl Zeiss, Oberkochen, Germany), and the fluorescence was quantified with ImageJ (National Institutes of Health, Bethesda, MD, USA). Intracellular fluorescence intensity/DAPI intensity was assessed as “relative fluorescence intensity”.

### 2.6. Western Blot Analysis

Cells were lysed with lysis buffer containing RIPA buffer (Biosolution, Seoul, Korea), protease inhibitor, and phosphatase inhibitor cocktail 2/3 (Sigma-Aldrich Chemicals, St. Louis, MO, USA). The protein concentration for each sample was quantified using a BCA protein assay kit (Thermo Fisher Scientific, Waltham, MA, USA) according to the manufacturer’s protocol. The proteins (50 µg) were separated and then transferred onto PVDF membranes. The samples were blocked for 1 h with 5% skim milk (BD, Franklin Lakes, NJ, USA). Membranes were then incubated with primary antibodies against ALP (Santa Cruz Biotechnology, Santa Cruz, CA, USA), COL1A1 (Abcam, Cambridge, UK), OCN (Santa Cruz Biotechnology, Santa Cruz, CA, USA), RUNX2 (Santa Cruz Biotechnology, Santa Cruz, CA, USA), PDLIM3 (Santa Cruz Biotechnology, Santa Cruz, CA, USA), and β-actin (Santa Cruz Biotechnology, Santa Cruz, CA, USA). Anti-rabbit IgG HRP-linked antibody (Cell Signaling Technology, Danvers, MA, USA) and m-IgGκ BP-HRP (Santa Cruz Biotechnology, Santa Cruz, CA, USA) antibodies were used as secondary antibodies. The specific information of the antibodies used in the study are listed below as Table 2. Chemi-Doc (Bio-Rad Laboratories, Hercules, CA, USA) was used to visualize the membranes with enhanced chemiluminescence detection reagent (GE Healthcare, Chicago, IL, USA).

### 2.7. Exosome Isolation

hADMSCs were cultured in hADMSCs growth medium until they were 80% confluent. The cell-cultured medium was then replaced with high-glucose DMEM (Gibco, Amarillo, TX, USA) containing 10% exosome-depleted fetal bovine serum (Gibco, Amarillo, TX, USA) and incubated for 3 days. Exosome isolation was performed using Total Exosome Isolation Reagent (Invitrogen, Waltham, MA, USA) and exosome concentration was quantified with a BCA protein assay kit (Thermo Fisher Scientific, Waltham, MA, USA) according to the protocols provided by the manufacturers.

### 2.8. THP-1 Cell Culture and Osteoclasts Differentiation

THP-1 human monocytes were purchased from ATCC (Manassas, VA, USA). Biological passage number 8 was used to culture the THP-1 cells in RPMI1640 (Welgene, Gyeongsan, Korea) containing 10% FBS, 2 mM L-glutamine, and 1% penicillin–streptomycin. Cells were seeded in 6-well plates (Nunc, Rochester, NY, USA) at the density of 3 × 10^5^ cells/well and differentiated into M0 macrophages using phorbol 12-myristate 13-acetate (Sigma-Aldrich Chemical, St. Louis, MO, USA) for 48 h. Then, the M0 macrophages were differentiated into osteoclasts in high-glucose DMEM (Gibco, Amarillo, TX, USA) containing 1% penicillin-streptomycin (Gibco, Amarillo, TX, USA), 10% fetal bovine serum (Alphabioregen, Boston, MA, USA), 50 ng/mL RANKL (R&D Systems, Minneapolis, MN, USA), and 50 ng/mL M-CSF (R&D Systems, Minneapolis, MN, USA) for 14 days. The differentiation medium was replaced with fresh medium every 2 days. TRAP-positive osteoclasts were validated using a TRAP staining kit (Cosmo Bio, Tokyo, Japan) according to the manufacturer’s protocol.

### 2.9. Coculture of hADMSCs with Differentiated Cells

hADMSCs were seeded in 6-well cell culture plates at the density of 6000 cells/cm^2^ and differentiated into adipocytes or osteoblasts for 21 days after the cells were 80% confluent. Another set of hADMSCs cells were seeded in 0.4 μm Transwell inserts (Corning Inc., Corning, NY, USA) at the density of 6000 cells/cm^2^ and incubated until they were 80% confluent. Then, the 0.4 μm Transwell inserts with hADMSCs of 80% confluency were added to the differentiated cells in 6-well cell culture plates and cocultured in low-glucose DMEM (Gibco, Amarillo, TX, USA) supplemented with 1% penicillin-streptomycin (Gibco, Amarillo, TX, USA), 10% fetal bovine serum (Alphabioregen, Boston, MA, USA) for 3 or 7 days.

### 2.10. Establishment of In Vitro Bone Matrix System with Osteoblasts and Osteoclasts

hADMSCs were seeded in 6-well cell culture plates at the density of 6000 cells/cm^2^ and differentiated into osteoblasts for 21 days after they were 80% confluent. THP-1 human monocytes were seeded in a 0.4 μm Transwell insert (Corning Inc., Corning, NY, USA) at a density of 3 × 10^5^ cells/well and differentiated into M0 macrophages using phorbol 12-myristate 13-acetate (Sigma-Aldrich Chemical, St. Louis, MO, USA) for 48 h. Then, the differentiation of M0 macrophages to osteoclasts was induced in high-glucose DMEM (Gibco, Amarillo, TX, USA) supplemented with the osteoclast differentiation agents described above for 14 days. The differentiation medium was replaced with fresh medium every 2 days. Finally, the 0.4 μm Transwell inserts with the differentiated osteoclasts were added to the 6-well cell culture plates with differentiated osteoblasts and cocultured in low-glucose DMEM (Gibco, Amarillo, TX, USA) supplemented with 1% penicillin-streptomycin (Gibco, Amarillo, TX, USA) and 10% fetal bovine serum (Alphabioregen, Boston, MA, USA) for 3 days.

### 2.11. Treatment of PD-L1 and hADMSCs Derived Exosomes

hADMSCs were seeded in 6-well cell culture plates at the density of 6000 cells/cm^2^ and differentiated into osteoblasts for 21 days after they were 80% confluent. THP-1 human monocytes were seeded at the density of 3 × 10^5^ cells/well and differentiated into M0 macrophages for 48 h, then differentiated into osteoclasts for 14 days. The cocultured environments (osteoblasts with hADMSCs, osteoblasts with osteoclasts) were established as mentioned above. PD-L1 (500 ng/mL) and exosome (10 µg/mL, 2.5 × 10^8^ particles/mL) treatment upon osteoblasts, cocultured osteoblasts with hADMSCs, and cocultured osteoblasts with osteoclasts were performed for 3 days.

### 2.12. Inhibition of the PD-1/PD-L1 Pathway

hADMSCs were seeded in 6-well cell culture plates at the density of 6000 cells/cm^2^ and differentiated into adipocytes or osteoblasts for 21 days after they were 80% confluent. THP-1 human monocytes were seeded at the density of 3 × 10^5^ cells/well and differentiated into M0 macrophages for 48 h, then differentiated into osteoclasts for 14 days. PD-1/PD-L1 axis inhibition was performed in monocultured adipocytes, cocultured adipocytes with hADMSCs, osteoblasts, cocultured osteoblasts with hADMSCs and cocultured osteoblasts with osteoclasts. PD-1/PD-L1 axis inhibition was performed to validate the function of the PD-1/PD-L1 axis. Cells were treated with 10 μM PD-1/PD-L1 axis inhibitor BMS202 (MedChemExpress, Princeton, NJ, USA) for 3 days to induce thermal stabilization of PD-L1 and block human PD-1/PD-L1 interactions.

### 2.13. Enzyme-Linked Immunosorbent Assay (ELISA)

Secretion levels of proinflammatory cytokines, TNF-α, and IL-1β in cell culture media of monocultured osteoblasts, cocultured osteoblasts with hADMSCs, and PD-L1 treated osteoblasts were determined using human TNF-α and an IL-1β, ELISA kit (R&D Systems, Minneapolis, MN, USA) according to the manufacturer’s instructions.

### 2.14. Nano Particle Tracking Analysis (NTA)

The isolated exosomes were diluted (1/200 dilution) in DPBS (Gibco, Amarillo, TX, USA) and 0.3 mL supernatant was loaded into the sample chamber of an LM10 unit (Nanosight, Amesbury, UK) The data were analyzed with a NanoSight NTA 3.4 analytical software unit (Nanosight, Amesbury, UK). A power of 55 mW and wavelength of 642 nm laser beam were used at 22 °C. A total of 100 control beads were used to calibrate the condition of the instrument.

### 2.15. Statistical Analysis

GraphPad Prism 5.0 (GraphPad Software Inc., San Diego, CA, USA) was used for all statistical analyses. Results are expressed as the mean ± standard error of at least three independent experiments, and the numbers of experiments (N) are specified on each figure. Differences were assessed with one-way ANOVA and Tukey’s test to account for multiple comparisons. Comparisons of only two samples were assessed with Student’s *t*-test.

## 3. Results

### 3.1. PD-1 Expression in Differentiated Cells Derived from hADMSCs

**Figure 1:**
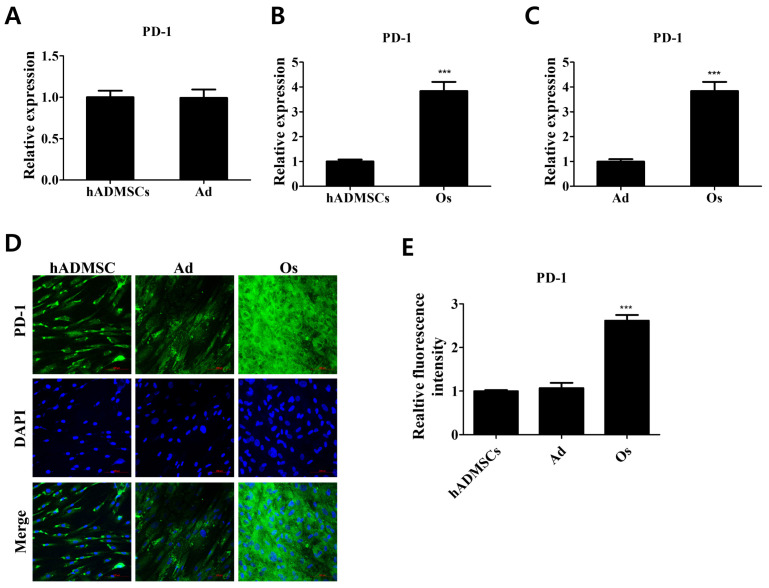
Relative PD-1 expression levels in differentiated cells derived from hADMSCs. (**A**) Relative mRNA expression level of *PD-1* in adipocytes (Ad) and (**B**) osteoblasts (Os) compared with hADMSCs (human adipose-derived mesenchymal stem cells). (**C**) Variance in the expression level of PD-1 in differentiated cells. *N* = 3 trials per sample and control. (**D**) Cells were stained with PD-1 (green); DAPI was used to stain the nuclei (blue). (**E**) Relative fluorescence intensity of PD-1 receptor on cell surface of differentiated cells. *N* = 5 trials per sample and control. Data are presented as mean ± SEM. *** *p* < 0.001, compared with the hADMSCs and Ad group. hADMSCs: human adipose-derived mesenchymal stem cells; Ad: adipocytes; Os: osteoblasts.

The expression levels of PD-1 in differentiated cells compared with those in hADMSCs were evaluated (Figure 1). Cells were differentiated into adipocytes or osteoblasts for 21 days in differentiation medium as described (Figure S1). The relative mRNA expression level of PD-1 was increased in osteoblasts compared to hADMSCs, whereas that of adipocytes showed no significant change (Figure 1A–C). The relative fluorescence intensity of PD-1 in osteoblasts compared with hADMSCs showed corresponding results, demonstrating that PD-1 expression varies with differentiation lineages and is significantly increased in osteogenic differentiated cells (Figure 1D,E). PD-1 is mostly expressed in immune cells, such as tumor-specific T cells, monocytes, and macrophages [20,22]. We first elucidated that PD-1 receptor expression increases upon osteogenic differentiation of hADMSCs, and our results suggest that the increased expression of PD-1 might be important for the regulation of osteogenic properties due to the reciprocal expression of the receptor in adipocytes and osteoblasts.

### 3.2. Immunosuppressive Effects Modulating the Expressions of Proinflammatory Cytokines in Differentiated Cells Cocultured with hADMSCs

**Figure 2:**
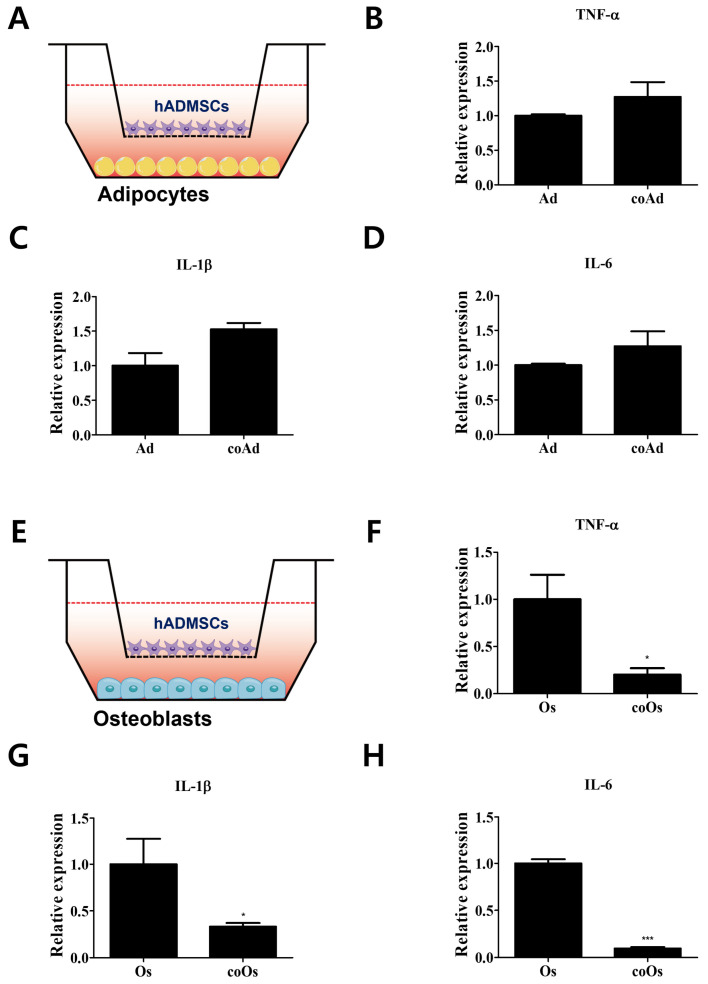
Regulation of the expression levels of proinflammatory cytokines in differentiated cells cocultured with hADMSCs. (**A**) Differentiated adipocytes were cocultured with hADMSCs. Relative mRNA expression levels of (**B**) *TNF-α*, (**C**) *IL-1β*, and (**D**) *IL-6*. (**E**) Differentiated osteoblasts were cocultured with hADMSCs. Relative mRNA expression levels of (**F**) *TNF-α*, (**G**) *IL-1β*, and (**H**) *IL-6*. *N* = 3 trials per sample and control. Data are presented as mean ± SEM. * *p* < 0.05 and *** *p* < 0.001, compared with the Os group. hADMSCs: human adipose-derived mesenchymal stem cells; Ad: adipocytes; coAd: cocultured adipocytes; Os: osteoblasts; coOS: cocultured osteoblasts.

It is well known that proinflammatory cytokines, including tumor necrosis factor-α (TNF-α), interleukin-1β (IL-1β), and interleukin-6 (IL-6), are produced by various cell types to upregulate inflammatory reactions [23,24,25,26]. Recent studies have shown that blocking PD-L1 promotes IL-6 and TNF-α expression in macrophages [27]. However, the immunosuppressive effects mediated by crosstalk between pro-inflammatory cytokines and the PD-1/PD-L1 axis in hADMSCs and their adjacent cells are yet to be understood. Here, cocultured environments of differentiated cells and hADMSCs were established and the expression levels of proinflammatory cytokines were evaluated to investigate the immunosuppressive effects in differentiated cells mediated by crosstalk with hADMSCs (Figure 2). The expression levels of *TNF-α*, *IL-1β*, and *IL-6*, which act as proinflammatory cytokines, showed no significant differences between cocultured and monocultured adipocytes (Figure 2A–D). However, osteoblasts cocultured with hADMSCs showed decreased proinflammatory cytokine expression levels compared with monocultured osteoblasts, reflecting the immunosuppressive potential of hADMSCs (Figure 2E–H). The secreted proinflammatory cytokine levels of TNF-α and IL-1β were decreased in the cell- cultured medium of cocultured osteoblasts with hADMSCs and PD-L1 treated osteoblasts compared with monocultured osteoblasts (Figure S2). That is, hADMSCs influenced the cocultured osteoblasts, thereby modulating immunosuppressive effects via paracrine actions through permeable membranes.

### 3.3. Attenuation of the Expression of Proinflammatory Cytokines by Inhibition of the PD-1/PD-L1 Axis in Differentiated Cells Cocultured with hADMSCs

**Figure 3:**
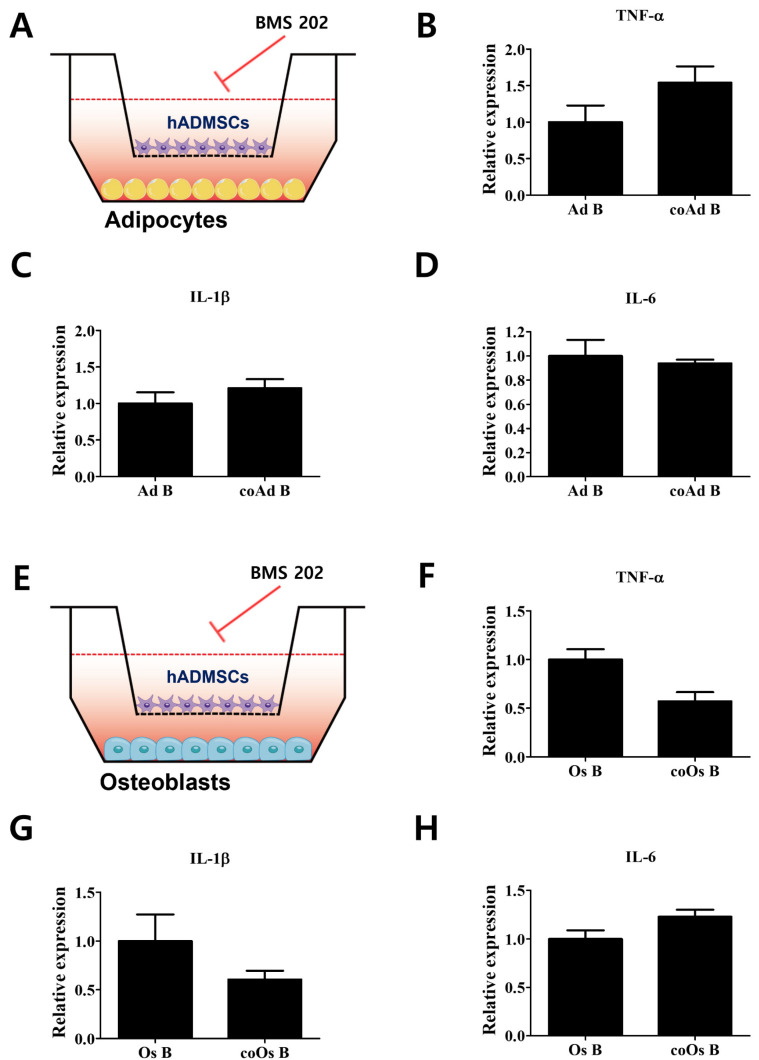
Attenuation of proinflammatory cytokine expression by PD-1/PD-L1 axis inhibition in differentiated cells cocultured with hADMSCs. (**A**) PD-1/PD-L1 interaction was inhibited in differentiated adipocytes cocultured with hADMSCs. Relative mRNA expression levels of (**B**) *TNF-α*, (**C**) *IL-1β*, and (**D**) *IL-6*. (**E**) PD-1/PD-L1 interaction was inhibited in differentiated osteoblasts cocultured with hADMSCs. Relative mRNA expression levels of (**F**) *TNF-α*, (**G**) *IL-1β*, and (**H**) *IL-6*. *N* = 3 trials per sample and control. Data are presented as mean ± SEM. hADMSCs: human adipose-derived mesenchymal stem cells; Ad: adipocytes; coAd: cocultured adipocytes; Os: osteoblasts; coOs: cocultured osteoblasts; B: BMS 202 (10 µM).

To examine whether the PD-1/PD-L1 axis is involved in the decrease in proinflammatory cytokine expression in cocultured osteoblasts, PD-1/PD-L1 interaction was inhibited by BMS 202 (Figure 3). Expression levels of *TNF-α*, *IL-1β*, and *IL-6* in cocultured adipocytes were maintained, even when PD-1/PD-L1 interaction was inhibited (Figure 3A–D). However, the decreased expression of proinflammatory cytokines in cocultured osteoblasts was attenuated upon inhibition of the PD-1/PD-L1 axis, after which no significant difference between cocultured and monocultured osteoblasts was detected (Figure 3E–H). We ruled out any effect which can affect the cells by showing the changes in the expression levels of proinflammatory cytokines upon BMS202 treatment in monocultured osteoblasts (Figure S3). The changes upon BMS treatment were not statistically significant compared with non-treated cells and confirmed that the action observed was via the inhibition of PD-1/PD-L1 axis. Taken together, we assume that the elevated expression of PD-1 in differentiated osteoblasts interacted with PD-L1 secreted by hADMSCs in cocultured environments, thereby eliciting an immunosuppressive response in osteoblasts.

### 3.4. Regulatory Effects of the PD-1/PD-L1 Axis on Osteogenic Properties of Osteoblasts Cocultured with hADMSCs

**Figure 4:**
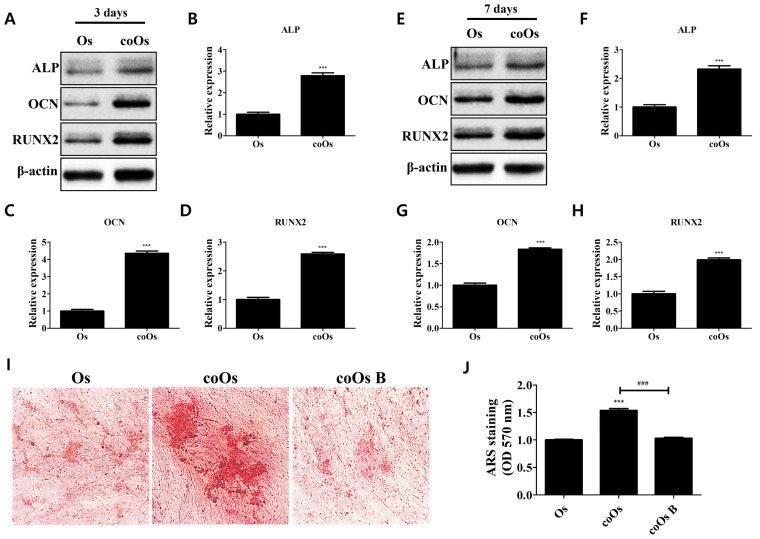
Effects of the PD-1/PD-L1 axis on osteogenic properties in osteoblasts cocultured with hADMSCs. (**A**) Expression levels of osteogenic marker proteins in monocultured or cocultured osteoblasts for 3 days. Relative expression levels of (**B**) ALP, (**C**) OCN, and (**D**) RUNX2 in cocultured osteoblasts for 3 days compared with monocultured osteoblasts. (**E**) Expression levels of osteogenic marker proteins in monocultured or cocultured osteoblasts for 7 days. Relative expression levels of (**F**) ALP, (**G**) OCN, and (**H**) RUNX2 in cocultured osteoblasts for 7 days compared with monocultured osteoblasts. *N* = 3 trials per sample and control. (**I**) Enhancement of matrix mineralization in cocultured osteoblasts and attenuation via inhibition of PD-1/PD-L1 interaction. (**J**) Stained calcium deposits were dissolved and quantified. *N* = 5 trials per sample and control. Data are presented as mean ± SEM. *** *p* < 0.001, compared with the Os group and ^###^ *p* < 0.001, compared with the coOs group. Os: osteoblasts; coOs: cocultured osteoblasts; B: BMS 202 (10 µM).

Recent studies have revealed that proinflammatory cytokine networks are involved in osteoblast differentiation and bone metabolism processes [28,29,30,31]; alkaline phosphatase (ALP), osteocalcin (OCN), and Runt-related transcription factor 2 (RUNX2) contribute to the osteogenic differentiation of osteogenic progenitor cells and are considered marker proteins of differentiated osteoblasts [28,32]. Here, osteogenic marker expressions in monocultured and cocultured osteoblasts and calcium deposits were evaluated (Figure 4). Compared with monocultured osteoblasts, those cocultured for 3 or 7 days both exhibited elevated expression of osteogenic markers (ALP, OCN, RUNX2), which contribute to the enhanced osteogenic properties (Figure 4A–H). Relative quantities of stained calcium deposits, which are the product of bone mineralization, were increased in cocultured compared with monocultured osteoblasts, and the increased calcium deposits were attenuated upon inhibition of PD-1/PD-L1 interaction, after which no significant difference with monocultured osteoblasts was observed, as indicated by decreased calcium deposits stained in cocultured osteoblasts treated with BMS 202 (Figure 4I,J). This result indicates that the PD-1/PD-L1 axis not only contributes to the anti-inflammatory action in osteoblasts but also enhances the osteogenic properties mediated by the interaction between osteoblasts and hADMSCs.

### 3.5. Effect of Exosomal PD-L1 on Bone Matrix Formation of Osteoblasts

**Figure 5:**
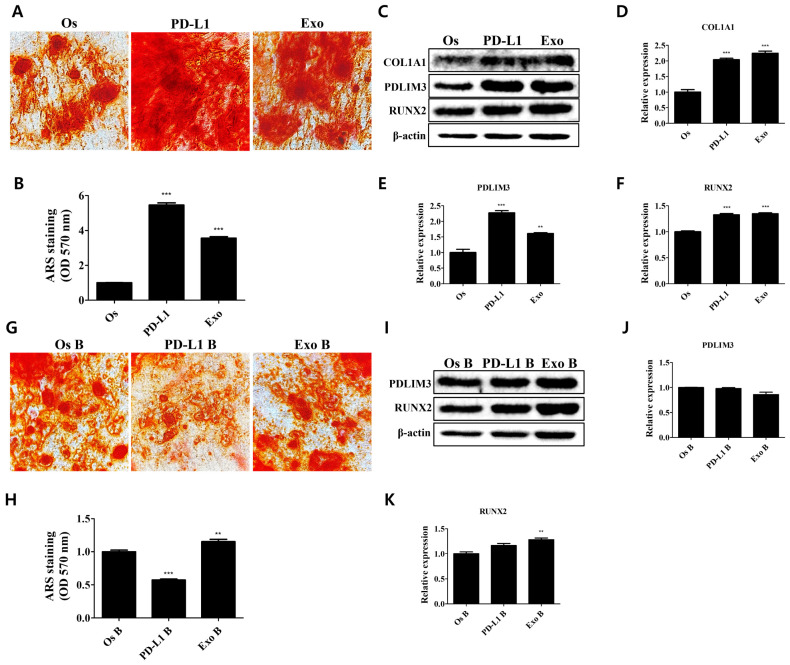
Regulatory effects of exosomal PD-L1 on bone matrix formation in osteoblasts. (**A**) Regulatory effects on the enhancement of matrix mineralization in PD-L1- and exosome-treated osteoblasts. (**B**) Stained calcium deposits were dissolved and quantified. *N* = 5 trials per sample and control. (**C**) Expression levels of proteins mediating the osteogenic properties upon PD-L1 and exosome treatment. Relative expression levels of (**D**) COL1A1, (**E**) PDLIM3, and (**F**) RUNX2 in osteoblasts. *N* = 3 trials per sample and control. (**G**) Attenuation of the enhanced matrix mineralization in PD-L1- and exosome-treated osteoblasts via blocking of PD-1/PD-L1 interaction. (**H**) Stained calcium deposits were dissolved and quantified. *N* = 5 trials per sample and control. (**I**) Expression levels of proteins mediating the osteogenic properties upon the inhibition of PD-1/PD-L1 interaction in PD-L1- and exosome-treated osteoblasts. Relative expression levels of (**J**) PDLIM3 and (**K**) RUNX2 in osteoblasts. *N* = 5 trials per sample and control. Data are presented as mean ± SEM. ** *p* < 0.01, and *** *p* < 0.001, compared with the Os and Os B group. Os: osteoblasts; PD-L1: PD-L1 (500 ng/mL); Exo: exosome (10 µg/mL, 2.5 × 10^8^ particles/mL); B: BMS 202 (10 µM).

To investigate the effect of the PD-1/PD-L1 axis on the bone matrix formation of osteoblasts, they were treated with PD-L1 and exosomes from hADMSCs (Figure 5). Studies have shown that PD-L1 is found in extracellular vesicles of certain cell types and that hMSCs profoundly secrete extracellular vesicles, which act as a modulatory factor of immune responses [8,13,14]. The exosomes derived from hADMSCs were characterized with nanoparticle tracking analysis (Figure S4). The concentration was quantified with a BCA protein assay. Our results show that the treatment with PD-L1 and exosomes from hADMSCs induced an increase in calcium deposits in treated compared with non-treated osteoblasts (Figure 5A,B). The expression levels of type 1 collagen (COL1A1), which forms the extracellular matrix of osteoblasts where the calcium deposits are located, and of PDZ and LIM domain protein 3 (PDLIM3), which is involved in collagen deposition, were profoundly increased upon both PD-L1 and exosome treatment (Figure 5C–E). Additionally, we observed a slight increase in the expression of RUNX2, which tends to be expressed in mature osteoblasts (Figure 5C,F). The inhibition of the PD-1/PD-L1 interaction diminished the increased calcium deposits and the expression of PDLIM3 and RUNX2 in both PD-L1- and exosome-treated osteoblasts (Figure 5 G–K). These results indicate that PD-L1 and the exosomes from hADMSCs interact with PD-1 in osteoblasts, thereby contributing to the enhanced bone matrix formation and osteogenic properties.

### 3.6. Regulatory Effect of the PD-1/PD-L1 Axis on Bone Homeostasis in Cocultured Environments

**Figure 6:**
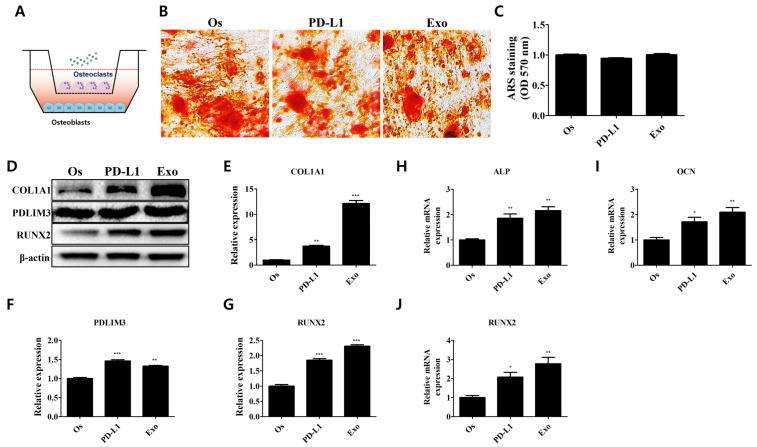
Modulatory effect of exosomal PD-L1 on bone matrix formation in cocultured environment. (**A**) Osteoblasts and osteoclasts were cocultured to mimic the bone metabolism under physiological conditions. (**B**) Maintenance of the bone homeostasis in PD-L1- and exosome-treated cocultured osteoblasts. (**C**) Stained calcium deposits were dissolved and quantified. *N* = 5 trials per sample and control. (**D**) Expression levels of proteins mediating the osteogenic properties upon PD-L1 and exosome treatment in cocultured osteoblasts. Relative expression levels of (**E**) COL1A1, (**F**) PDLIM3, and (**G**) RUNX2. *N* = 3 trials per sample and control. Relative mRNA expressions of osteogenic markers (**H**) *ALP*, (**I**) *OCN*, and (**J**) *RUNX2*. *N* = 3 trials per sample and control. Data are presented as mean ± SEM. * *p* < 0.05, ** *p* < 0.01, and *** *p* < 0.001, compared with the Os group. Os: osteoblasts (cocultured); PD-L1: PD-L1 (500 ng/mL); Exo: exosome (10 µg/mL, 2.5 × 10^8^ particles/mL).

**Figure 7:**
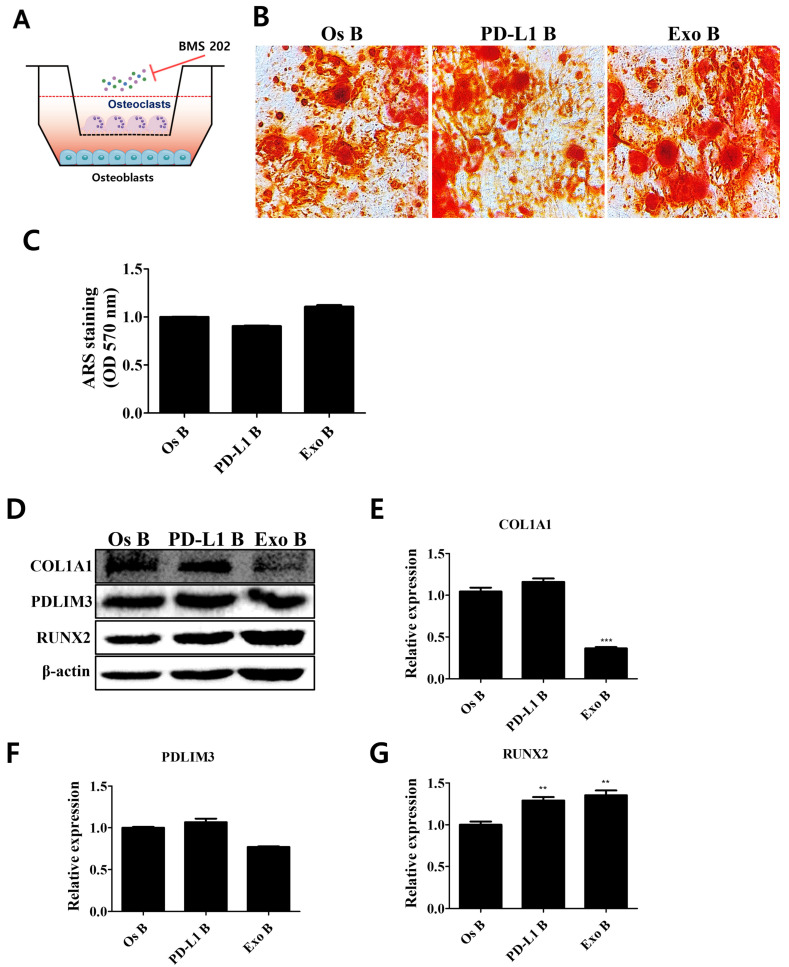
Attenuation of osteogenic properties in cocultured osteoblasts via inhibition of PD-1/PD-L1 interaction. (**A**) PD-1/PD-L1 interaction was inhibited in the cocultured environment. (**B**) Maintenance of bone mineralization in cocultured osteoblasts. (**C**) Stained calcium deposits were dissolved and quantified. *N* = 5 trials per sample and control. (**D**) Attenuation of the expression levels of proteins mediating osteogenic properties upon inhibition of PD-1/PD-L1 interaction. Relative expression levels of (**E**) COL1A1, (**F**) PDLIM3, and (**G**) RUNX2. *N* = 3 trials per sample and control. Data are presented as mean ± SEM. ** *p* < 0.01, and *** *p* < 0.001, compared with the Os B group. Os: osteoblasts (cocultured); PD-L1: PD-L1 (500 ng/mL); Exo: exosome (10 µg/mL, 2.5 × 10^8^ particles/mL); B: BMS 202 (10 µM).

Bone is extremely complex and a dynamic organ that is constantly remodeled by osteoblast and osteoclast crosstalk [19]. The balance between bone formation and bone resorption that needs to be maintained by osteoblasts and osteoclasts is crucial for the homeostasis of healthy bone [33]. The THP-1 monocytes were differentiated into osteoclasts and validated by their morphology and TRAP expression (Figure S5). We established a cocultured system of osteoblasts derived from hADMSCs and osteoclasts from THP-1 monocytes and evaluated the regulatory effects of the PD-1/PD-L1 axis on our in vitro bone metabolism system (Figure 6). Interestingly, PD-L1- or hADMSC-derived exosome treatment in our cocultured system did not enhance the calcium deposits in the osteoblast-mediated bone matrix, which is inconsistent with our results depicted in Figure 5 (Figure 6A–C). However, the expression levels of molecular factors related to the osteogenic properties of osteoblasts, such as COL1A1, PDLIM3, and RUNX2, were increased upon PD-L1 or exosome treatment in our cocultured system (Figure 6D–G). Similarly, osteogenic marker expression (*ALP*, *OCN*, *RUNX2*) levels were also increased upon PD-L1 and exosome treatment in osteoblasts cocultured with osteoclasts (Figure 6H–J). The inhibition of PD-1/PD-L1 interaction did not affect the calcium deposits in the bone matrix, but the expression of COL1A1, which directly forms the bone matrix, was attenuated in PD-L1/exosome-treated osteoblasts (Figure 7). These results demonstrate that, unlike in the monocultured environment of osteoblasts, the PD-1/PD-L1 axis contributes to the increased expression of the factors related to the increased osteogenic properties of osteoblasts and maintains the balance between osteoblasts and osteoclasts in our in vitro bone metabolism model, where it mediates the bone homeostasis.

## 4. Discussion

hMSCs, the multipotent stem cells that can be differentiated into cells of multiple lineages, also possess great immunomodulatory properties; efforts are therefore being made to develop a hMSC-based cell therapy [34,35]. Considering the potential of hMSCs for regenerative medicine, it is important to improve their immunosuppressive effects and accelerate hMSC-mediated tissue regeneration to improve the overall efficacy of cell therapy [36]. PD-L1 is a type 1 transmembrane protein that is expressed in various cell types [37]. It binds to its receptor PD-1 and has immunoinhibitory functions, such as the inhibition of T cell responses [38]. There are studies that underline the functions of the PD-1/PD-L1 axis, but they are limited to cell therapies utilizing the immunomodulatory properties of the PD-1/PD-L1 axis [12,39]. Likewise, PD-L1 is profoundly expressed in hMSCs but earlier research on the modulatory function of the PD-1/PD-L1 axis has been confined to the immunosuppressive functions of hMSCs and their adjacent cells [9,40]. Here, we first demonstrate the potential of the PD-1/PD-L1 axis as a novel molecular target in stem cell therapy for bone regeneration and the immunomodulation of hMSCs.

We examined the expression levels of PD-1, the receptor of PD-L1, in differentiated adipocytes and osteoblasts from hADMSCs. Interestingly, our results reveal that PD-1 expression was profoundly increased only in osteoblasts. This raises the possibility that hADMSCs interact and mediate the immunosuppressive actions in osteoblasts via the PD-1/PD-L1 axis. To examine whether the PD-1/PD-L1 axis contributes to the interactions between hADMCs and differentiated cells, adipocytes, and osteoblasts were also cocultured with hADMSCs, and only the cocultured (not the monocultured) osteoblasts exhibited a decrease in the expression levels of proinflammatory cytokines, due to the increased expression of PD-1. Cocultured adipocytes did not exhibit any quantitative changes in the expression levels of proinflammatory cytokines. Upon inhibition of the PD-1/PD-L1 axis, the immunosuppressive responses in the coculture of osteoblasts with hADMCs diminished.

It has recently been found that cytokine networks are closely related to osteogenic properties in osteoblasts [28]. IL-6, TNF-α, and IL-1β are known to inhibit osteogenesis, matrix mineralization, and the expressions of osteogenic factors, including ALP, OCN, RUNX2, and COL1A1 [28,29,30,31]. Osteoblasts cocultured with hADMSCs for 3 or 7 days exhibited an increase in the expressions of osteogenic markers (ALP, OCN, RUNX2), as predicted. Calcium deposits were increased in cocultured osteoblasts compared with monocultured osteoblasts, and the increase diminished when the PD-1/PD-L1 axis was inhibited. These findings clarify that PD-L1 produced from hADMSCs not only contributes to the immunosuppressive effects but also enhances osteogenic properties in osteoblasts.

PD-L1 is secreted in extracellular form, such as exosomes, by tumor cells to modulate immune responses [12,13]. Recently, hMSCs have been found to be a source of exosomal PD-L1 contributing to enhanced immunomodulation [9,14]. We hypothesize that the immunosuppressive effects and regulation of osteogenic properties observed in our cocultured osteoblasts stem from the secretion of PD-L1 by hADMSCs. PD-L1 and hADMSCs-derived exosome treatment increased the calcium deposits in osteoblasts, whereas RUNX2, which is profoundly expressed in mature osteoblasts, was increased upon PD-L1 and exosome treatment. Osteoblasts are known to secrete large amounts of COL1A1 as a major component of the bone matrix [41]. COL1A1 is a key factor for calcium deposits, since calcification occurs on the collagen fiber matrix, crystalizing the tissue, and collagen deposition is known to be regulated by PDLIM3 [42,43,44]. The increased calcium deposits in osteoblasts were evidenced by elevated expression levels of COL1A1 and PDLIM3 upon PD-L1 and exosome treatment. Inhibition of PD-1/PD-L1 interaction diminished the increased osteogenic properties in osteoblasts, which illustrates that the increased osteogenic properties upon PD-L1 and exosome treatments were due to the PD-1/PD-L1 axis in osteoblasts.

Bone is constantly rejuvenated by dynamic interactions between osteoblasts and osteoclasts [33,45], and several studies have shown the successful establishment of bone metabolism models in vitro with osteoblasts and osteoclasts [18,46]. Here, we successfully established an in vitro cocultured model with hADMSC-derived osteoblasts and TRAP-positive osteoclasts differentiated from THP-1 monocytes. Interestingly, and different from our findings in monocultured osteoblasts, PD-L1 and exosome treatment in cocultured environments did not enhance the calcium deposits in osteoblasts. However, COL1A1 and PDLIM3 expression levels increased upon PD-L1 and exosome treatment. The increase in the expression levels of these factors involved in bone matrix formation diminished upon PD-1/PD-L1 inhibition, and the calcium deposits were found to be maintained, compared with non-treated cells. Osteoclasts originate from immune cells such as the monocytes–macrophages series, and the PD-1/PD-L1 axis is known to contribute to osteoclast formation and activity [19,20,21]. Homeostasis during the bone remodeling process mediated by bone formation and resorption by osteoblasts and osteoclasts is important. If an imbalance occurs between bone formation and resorption, the bone structure becomes susceptible to osteoporosis and osteopetrosis [47]. The calcium deposits in cocultured osteoblasts are formed as results from the interplay between osteoblasts (bone formation) and osteoclasts (bone resorption) [48,49]. A recent study showed that the PD-1/PD-L1 axis positively regulates osteoclast functions, including TRAP activity and differentiation of osteogenic precursor cells [20]. Based on the information and results, we assumed that the PD-1/PD-L1 axis positively regulated osteogenic properties in osteoblasts, and the PD-1/PD-L1 axis may contribute to the properties of osteoclasts, resulting in the maintenance of the calcium deposition levels in PD-L1- and exosome-treated cocultured osteoblasts in our cocultured system, which means that the PD-1/PD-L1 axis may simultaneously accelerate bone formation and resorption, maintaining bone homeostasis.

Overall, our study shows that PD-1 expression in osteoblasts contributes to immunosuppressive effects and osteogenic properties via the PD-1/PD-L1 axis. We found that the PD-1/PD-L1 axis in osteoblasts was regulated by hADMSC-derived exosomes in our cocultured system with hADMSCs. PD-L1 separately enhanced osteogenic properties, including bone mineralization and COL1A1 expression, in osteoblasts. However, PD-L1 rather contributed to bone homeostasis of osteoblasts in our cocultured system of osteoblasts and osteoclasts. These results provide new insights into molecular therapeutic targets for the prevention of bone diseases via the PD-1/PD-L1 axis.

## 5. Conclusions

In this study, we demonstrate how the PD-1/PD-L1 axis modulates immunosuppressive effects and bone homeostasis in osteoblasts differentiated from hADMSCs. We found that the PD-1/PD-L1 axis contributed to enhanced bone mineralization only in monocultured environments without osteoclasts. Our coculture system reveals that the PD-1/PD-L1 axis affecting both osteoblasts and osteoclasts contributes to the maintenance of bone homeostasis. These data provide fundamental information on the novel insights into the PD-1/PD-L1 axis that can be utilized as a therapeutic target in stem cell therapy in the prevention of bone diseases and therefore shed light on some of the challenges that remain in stem cell therapy, especially regarding the increase in the efficacy of therapeutic approaches.

## Figures and Tables

**Table 1:** List of oligonucleotide primers used in the study.

Gene Name		Sequences
PD-1	Forward	CCCTGGTGGTTGGTGTCGT
	Reverse	GCCTGGCTCCTATTGTCCCTC
TNF-α	Forward	AGAGAAGCCAACTACAGACC
	Reverse	CAGTATGTGAGAGGAAGAGAA
IL-6	Forward	CAGAACAGATTTGAGAGTAGTGA
	Reverse	CGCAGAATGAGATGAGTTGT
IL-1β	Forward	GGCTTATTACAGTGGCAATG
	Reverse	TAGTGGTGGTCGGAGATT
ALP	Forward	TCATCCGTGGTTGTATCA
	Reverse	GTGGTCTCAGTAGCATCT
RUNX2	Forward	AAGGCAGTTACATATCAATACAG
	Reverse	GAGGCAGAGGCTTCAATA
OCN	Forward	CGGAGTAGTCATCATTGTG
	Reverse	CGAGTGTTCATTCTGTTCA
FABP4	Forward	TCAAGAGCACCATAACCTT
	Reverse	TTCCACCACCAGTTTATCA
Adiponectin	Forward	ACCACTATGATGGCTCCACT
	Reverse	GGTGAAGAGCATAGCCTTGT
PPARγ	Forward	CGAAGACATTCCATTCACAA
	Reverse	CACAGACACGACATTCAAT
GAPDH	Forward	TATGACAACAGCCTCAAGAT
	Reverse	GAGTCCTTCCACGATACC

**Table 2:** List of antibodies used in the study.

Target	Catalogue Number	Dilution
ALP	SC-271431	1/500
COL1A1	Ab34710	1/500
OCN	SC-7449	1/500
RUNX2	SC-10758	1/500
PDLIM3	SC-98652	1/500
β-actin	SC-47778	1/500
PD-1	AF1086	1/200
Anti-rabbit IgG HRP-linked antibody	7074S	1/1500
m-IgGκ BP-HRP	SC-516102	1/1500
Donkey anti-goat IgG NorthernLights	NL003	1/1000

## Data Availability

The data presented in this study are contained within the article.

## Supplementary Materials

The following supporting information can be downloaded at: https://www.mdpi.com/article/10.3390/cells11193152/s1, Figure S1: Validation of the differentiated cells from hADMSCs; Figure S2: Regulation of the secreted levels of proinflammatory cytokines in differentiated osteoblasts cocultured with hADMSCs; Figure S3: Effect of PD-1/PD-L1 inhibition on monocultured osteoblasts; Figure S4: Characterization of exosomes derived from hADMSCs; Figure S5: Establishment of osteoclast differentiation from THP-1 monocytes.

## References

[1] Fu X., Liu G., Halim A., Ju Y., Luo Q., Song G. (2019). Mesenchymal Stem Cell Migration and Tissue Repair. Cells.

[2] Lindroos B., Suuronen R., Miettinen S. (2011). The Potential of Adipose Stem Cells in Regenerative Medicine. Stem Cell Rev. Rep..

[3] Sohni A., Verfaillie C.M. (2013). Mesenchymal Stem Cells Migration Homing and Tracking. Stem Cells Int..

[4] Liu S., Liu F., Zhou Y., Jin B., Sun Q., Guo S. (2020). Immunosuppressive Property of MSCs Mediated by Cell Surface Receptors. Front. Immunol..

[5] Lee S.C., Lee Y.J., Choi I., Kim M., Sung J.S. (2021). CXCL16/CXCR6 Axis in Adipocytes Differentiated from Human Adipose Derived Mesenchymal Stem Cells Regulates Macrophage Polarization. Cells.

[6] Lee S.-C., Lee Y.-J., Shin M.K., Sung J.-S. (2020). Regulation of CXCR6 Expression on Adipocytes and Osteoblasts Differentiated from Human Adipose Tissue-Derived Mesenchymal Stem Cells. Stem Cells Int..

[7] Bae S.J., Kim H.J., Won H.Y., Min Y.K., Hwang E.S. (2017). Acceleration of osteoblast differentiation by a novel osteogenic compound, DMP-PYT, through activation of both the BMP and Wnt pathways. Sci. Rep..

[8] Han Y., Yang J., Fang J., Zhou Y., Candi E., Wang J., Hua D., Shao C., Shi Y. (2022). The secretion profile of mesenchymal stem cells and potential applications in treating human diseases. Signal Transduct. Target. Ther..

[9] Davies L.C., Heldring N., Kadri N., Le Blanc K. (2017). Mesenchymal Stromal Cell Secretion of Programmed Death-1 Ligands Regulates T Cell Mediated Immunosuppression. Stem Cells.

[10] Han Y., Liu D., Li L. (2020). PD-1/PD-L1 pathway: Current researches in cancer. Am. J. Cancer Res..

[11] Salmaninejad A., Khorramshahi V., Azani A., Soltaninejad E., Aslani S., Zamani M.R., Zal M., Nesaei A., Hosseini S.M. (2018). PD-1 and cancer: Molecular mechanisms and polymorphisms. Immunogenetics.

[12] Zhou K., Guo S., Li F., Sun Q., Liang G. (2020). Exosomal PD-L1: New Insights Into Tumor Immune Escape Mechanisms and Therapeutic Strategies. Front. Cell Dev. Biol..

[13] Chen G., Huang A.C., Zhang W., Zhang G., Wu M., Xu W., Yu Z., Yang J., Wang B., Sun H. (2018). Exosomal PD-L1 contributes to immunosuppression and is associated with anti-PD-1 response. Nature.

[14] Li M., Soder R., Abhyankar S., Abdelhakim H., Braun M.W., Trinidad C.V., Pathak H.B., Pessetto Z., Deighan C., Ganguly S. (2021). WJMSC-derived small extracellular vesicle enhance T cell suppression through PD-L1. J. Extracell. Vesicles.

[15] Alonso-Goulart V., Ferreira L.B., Duarte C.A., de Lima I.L., Ferreira E.R., de Oliveira B.C., Vargas L.N., de Moraes D.D., Silva I.B.B., Faria R.D.O. (2018). Mesenchymal stem cells from human adipose tissue and bone repair: A literature review. Biotechnol. Res. Innov..

[16] Storti G., Scioli M.G., Kim B.-S., Orlandi A., Cervelli V. (2019). Adipose-Derived Stem Cells in Bone Tissue Engineering: Useful Tools with New Applications. Stem Cells Int..

[17] Delgado-Calle J., Riancho J.A. (2012). The Role of DNA Methylation in Common Skeletal Disorders. Biology.

[18] Owen R., Reilly G.C. (2018). In vitro Models of Bone Remodelling and Associated Disorders. Front. Bioeng. Biotechnol..

[19] Martin T.J., Ng K.W. (1994). Mechanisms by which cells of the osteoblast lineage control osteoclast formation and activity. J. Cell. Biochem..

[20] Wang K., Gu Y., Liao Y., Bang S., Donnelly C., Chen O., Tao X., Mirando A.J., Hilton M.J., Ji R.-R. (2020). PD-1 blockade inhibits osteoclast formation and murine bone cancer pain. J. Clin. Investig..

[21] Zuo H., Wan Y. (2022). Inhibition of myeloid PD-L1 suppresses osteoclastogenesis and cancer bone metastasis. Cancer Gene Ther..

[22] Wang X., Yang X., Zhang C., Wang Y., Cheng T., Duan L., Tong Z., Tan S., Zhang H., Saw P.E. (2020). Tumor cell-intrinsic PD-1 receptor is a tumor suppressor and mediates resistance to PD-1 blockade therapy. Proc. Natl. Acad. Sci. USA.

[23] Watkins L.R., Maier S.F., Goehler L.E. (1995). Immune activation: The role of pro-inflammatory cytokines in inflammation, illness responses and pathological pain states. Pain.

[24] Ayoub S., Berbéri A., Fayyad-Kazan M. (2020). Cytokines, Masticatory Muscle Inflammation, and Pain: An Update. J. Mol. Neurosci..

[25] Zhang J.M., An J. (2007). Cytokines, inflammation, and pain. Int. Anesthesiol. Clin..

[26] Umare V., Pradhan V., Nadkar M., Rajadhyaksha A., Patwardhan M., Ghosh K.K., Nadkarni A.H. (2014). Effect of Proinflammatory Cytokines (IL-6, TNF-*α*, and IL-1*β*) on Clinical Manifestations in Indian SLE Patients. Mediat. Inflamm..

[27] Liu S., Mi J., Liu W., Xiao S., Gao C. (2019). Blocking of checkpoint receptor PD-L1 aggravates osteoarthritis in macrophage-dependent manner in the mice model. Int. J. Immunopathol. Pharmacol..

[28] Amarasekara D., Kim S., Rho J. (2021). Regulation of Osteoblast Differentiation by Cytokine Networks. Int. J. Mol. Sci..

[29] Metzger C.E., Narayanan S.A. (2019). The Role of Osteocytes in Inflammatory Bone Loss. Front. Endocrinol..

[30] Lacey D., Simmons P., Graves S., Hamilton J. (2009). Proinflammatory cytokines inhibit osteogenic differentiation from stem cells: Implications for bone repair during inflammation. Osteoarthr. Cartil..

[31] Kaneshiro S., Ebina K., Shi K., Higuchi C., Hirao M., Okamoto M., Hashimoto J. (2014). IL-6 negatively regulates osteoblast differentiation through the SHP2/MEK2 and SHP2/Akt2 pathways in vitro. J. Bone Miner. Metab..

[32] Lehti M.S., Henriksson H., Rummukainen P., Wang F., Uusitalo-Kylmälä L., Kiviranta R., Heino T., Kotaja N., Sironen A. (2018). Cilia-related protein SPEF2 regulates osteoblast differentiation. Sci. Rep..

[33] Kim J.-M., Lin C., Stavre Z., Greenblatt M.B., Shim J.-H. (2020). Osteoblast-Osteoclast Communication and Bone Homeostasis. Cells.

[34] Moll G., Hoogduijn M.J., Ankrum J.A. (2020). Editorial: Safety, Efficacy and Mechanisms of Action of Mesenchymal Stem Cell Therapies. Front. Immunol..

[35] Wu X., Jiang J., Gu Z., Zhang J., Chen Y., Liu X. (2020). Mesenchymal stromal cell therapies: Immunomodulatory properties and clinical progress. Stem Cell Res. Ther..

[36] Lee B.-C., Kang K.-S. (2020). Functional enhancement strategies for immunomodulation of mesenchymal stem cells and their therapeutic application. Stem Cell Res. Ther..

[37] Wen M., Cao Y., Wu B., Xiao T., Cao R., Wang Q., Liu X., Xue H., Yu Y., Lin J. (2021). PD-L1 degradation is regulated by electrostatic membrane association of its cytoplasmic domain. Nat. Commun..

[38] Akinleye A., Rasool Z. (2019). Immune checkpoint inhibitors of PD-L1 as cancer therapeutics. J. Hematol. Oncol..

[39] Ye L., Zhu Z., Chen X., Zhang H., Huang J., Gu S., Zhao X. (2021). The Importance of Exosomal PD-L1 in Cancer Progression and Its Potential as a Therapeutic Target. Cells.

[40] Di Tinco R., Bertani G., Pisciotta A., Bertoni L., Pignatti E., Maccaferri M., Bertacchini J., Sena P., Vallarola A., Tupler R. (2021). Role of PD-L1 in licensing immunoregulatory function of dental pulp mesenchymal stem cells. Stem Cell Res. Ther..

[41] Blair H.C., Larrouture Q.C., Li Y., Lin H., Beer-Stoltz D., Liu L., Nelson D.J. (2017). Osteoblast Differentiation and Bone Matrix Formation In Vivo and In Vitro. Tissue Eng. B Rev..

[42] Bourne L.E., Wheeler-Jones C.P., Orriss I.R. (2021). Regulation of mineralisation in bone and vascular tissue: A comparative review. J. Endocrinol..

[43] Lodder E.M., Scicluna B.P., Beekman L., Arends D., Moerland P.D., Tanck M.W., Adriaens M.E., Bezzina C.R. (2014). Integrative Genomic Approach Identifies Multiple Genes Involved in Cardiac Collagen Deposition. Circ. Cardiovasc. Genet..

[44] Wang D., Fang J., Lv J., Pan Z., Yin X., Cheng H., Guo X. (2019). Novel polymorphisms in PDLIM3 and PDLIM5 gene encoding Z-line proteins increase risk of idiopathic dilated cardiomyopathy. J. Cell Mol. Med..

[45] Salhotra A., Shah H.N., Levi B., Longaker M.T. (2020). Mechanisms of bone development and repair. Nat. Rev. Mol. Cell Biol..

[46] Borciani G., Montalbano G., Baldini N., Cerqueni G., Vitale-Brovarone C., Ciapetti G. (2020). Co–culture systems of osteoblasts and osteoclasts: Simulating in vitro bone remodeling in regenerative approaches. Acta Biomater..

[47] Jimi E., Hirata S., Osawa K., Terashita M., Kitamura C., Fukushima H. (2012). The Current and Future Therapies of Bone Regeneration to Repair Bone Defects. Int. J. Dent..

[48] Schröder H., Wang X., Wiens M., Diehl-Seifert B., Kropf K., Schloßmacher U., Müller W. (2012). Silicate modulates the cross-talk between osteoblasts (SaOS-2) and osteoclasts (RAW 264.7 cells): Inhibition of osteoclast growth and differentiation. J. Cell. Biochem..

[49] Remmers S.J.A., de Wildt B.W.M., Vis M.A.M., Spaander E.S.R., de Vries R.B.M., Ito K., Hofmann S. (2021). Osteoblast-osteoclast co-cultures: A systematic review and map of available literature. PLoS ONE.

