# Supplementary material for: Immunomodulatory Effect and Bone Homeostasis Regulation in Osteoblasts Differentiated from hADMSCs via the PD-1/PD-L1 Axis
Source: Cells. 2022 Oct 7;11(19):3152. doi: 10.3390/cells11193152 (PMC9564087; doi:10.3390/cells11193152)
Supplement: Supplementary file 1 [file cells-11-03152-s001.zip › Supplementarry file S1.pdf]

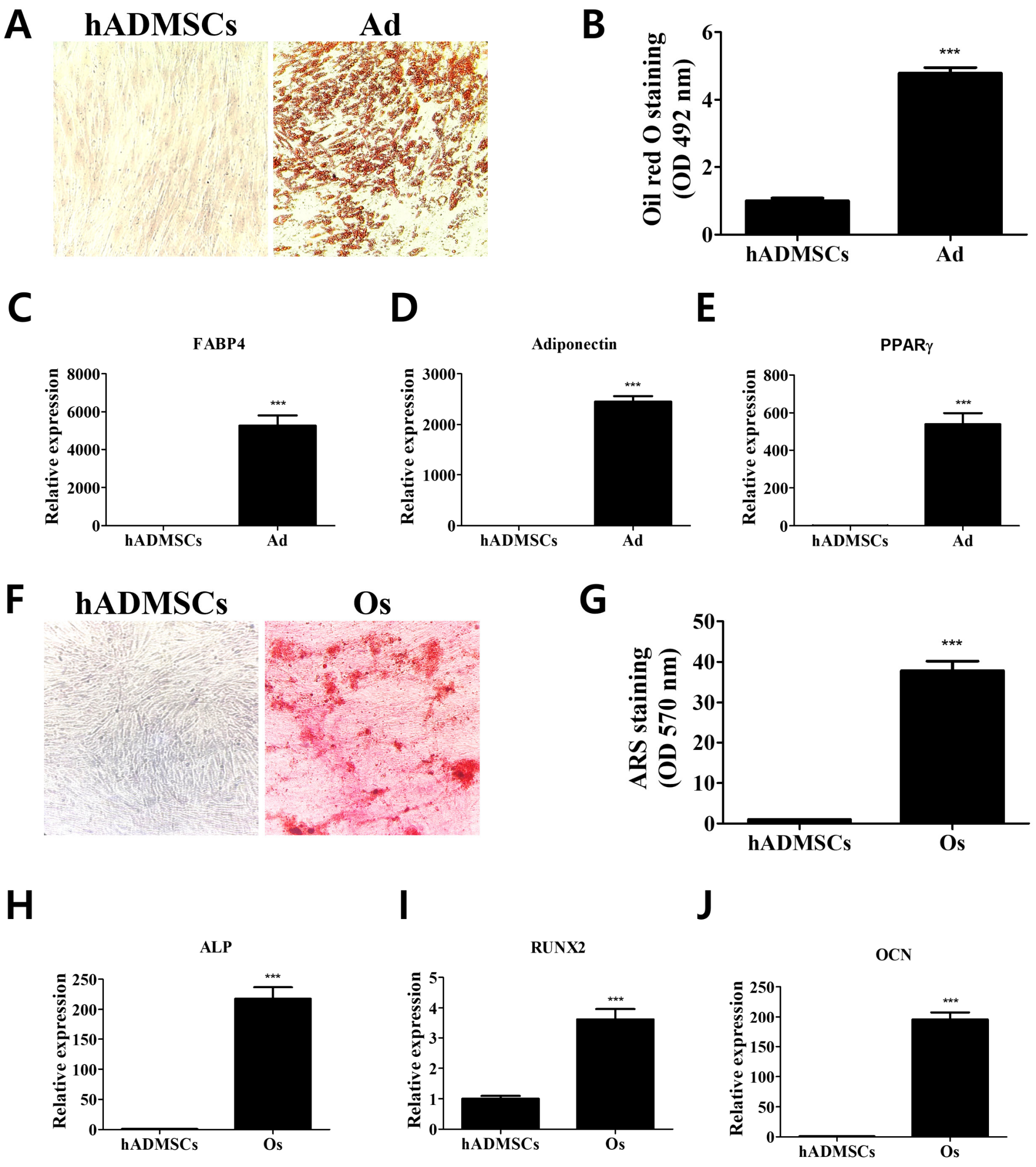

Figure S1. Validation of the differentiated cells from hADMSCs. (**A**) Oil Red O stained of hADMSCs and Adipocytes. (**B**) Stained lipid droplets are dissolved and quantified. $N = 5$ trials per sample and control. Relative expression of adipocyte specific markers (**C**) *FABP4*, (**D**) *Adiponectin* and (**E**) *PPARγ*. $N = 3$ trials per sample and control. (**F**) Alizarin Red S stained hADMSCs and osteoblasts. (**G**) Stained calcium deposits were dissolved and quantified. $N = 5$ trials per sample and control. Relative expression of osteoblast specific markers (**H**) *ALP*, (**I**) *OCN* and (**J**) *RUNX2*. $N = 3$ trials per sample and control. Data are presented as mean ± SEM. *** $p < 0.001$, compared with the hADMSCs group. Ad: adipocytes; Os: osteoblasts.

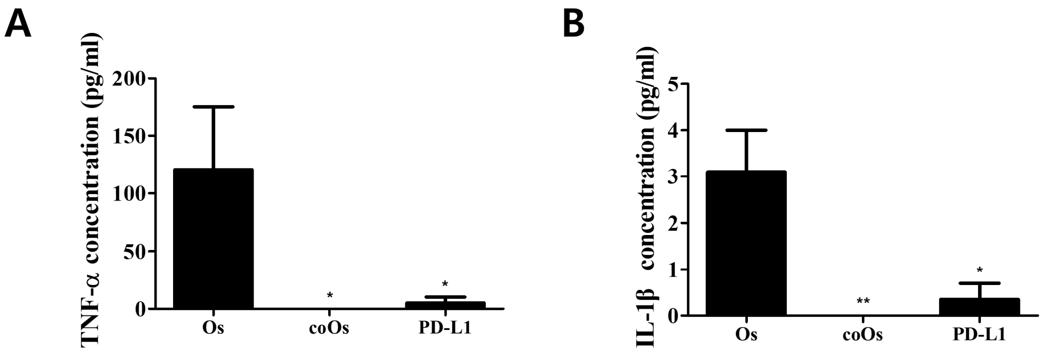

Figure S2. Regulation of the secreted levels of proinflammatory cytokines in differentiated osteoblasts cocultured with hADMSCs. Secreted levels of proinflammatory cytokines (**A**) TNF-α, (**B**) IL-1β in cell culture medium. $N = 3$ trials per sample. Data are presented as mean ± SEM. * $p < 0.05$ and ** $p < 0.01$ compared with the Os group. Os: osteoblasts; coOs: cocultured osteoblasts; PD-L1: PD-L1 treated monocultured osteoblasts (500 ng/ml).

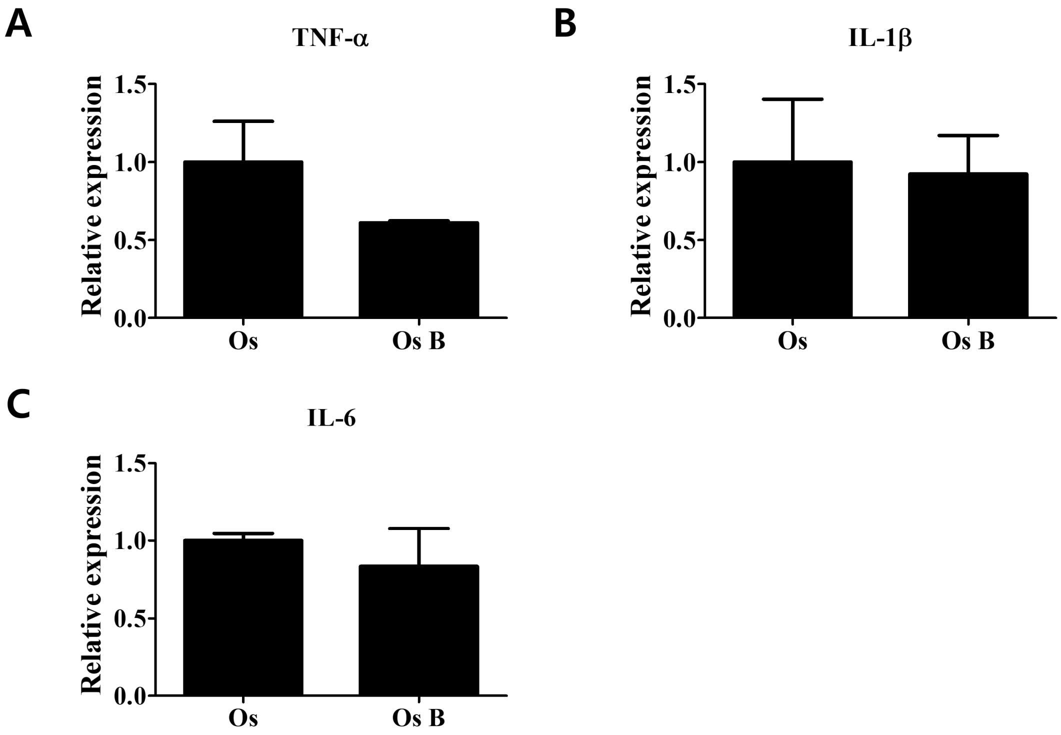

Figure S3. Effect of PD-1/PD-L1 inhibition on monocultured osteoblasts. Relative mRNA expression levels of proinflammatory cytokines (**A**) *TNF-α*, (**B**) *IL-1β*, and (**C**) *IL-6*. *N* = 3 trials per sample. Data are presented as mean ± SEM. Os: osteoblasts; B: BMS 202 (10 μM).

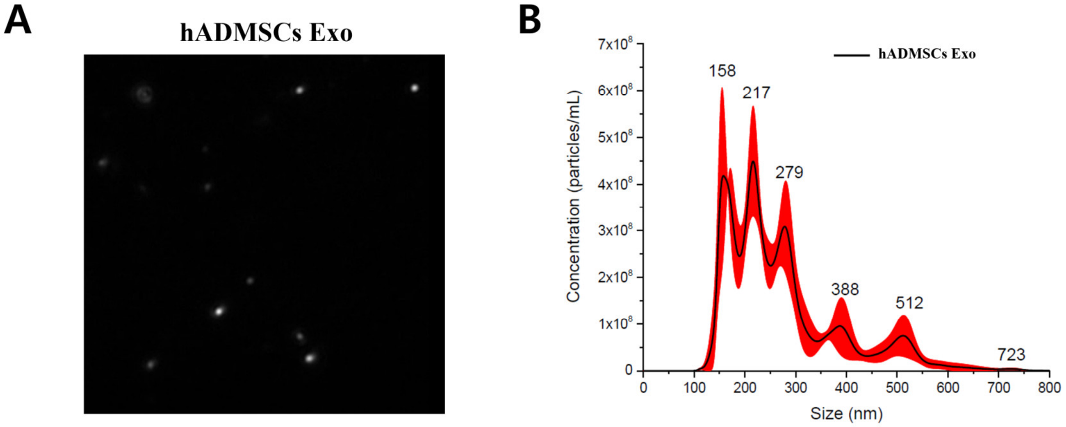

Figure S4. Characterization of exosomes dervied from hADMSCs. (**A**) Nanoparticle tracking analysis (NTA) image of hADMSCs derived exosomes. (**B**) Number concentration profiles of exosomes from hADMSCs. *N* = 3 trials per sample and control. hADMSCs Exo: hADMSCs derived exosome.

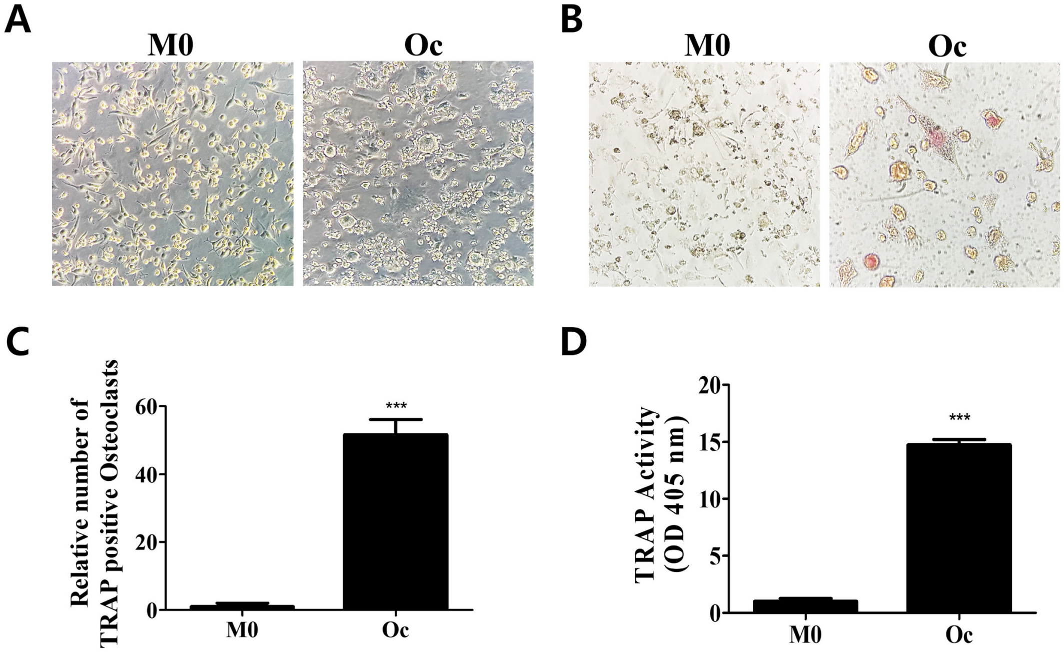

Figure S5. Establishment of osteoclast differentiation from THP-1 monocytes. (**A**) Morphological difference between M0 macrophages and differentiated osteoclasts. $N = 3$ trials per sample and control. (**B**) TRAP stained M0 macrophages and osteoclasts. (**C**) Relative number of TRAP positive osteoclasts. $N = 3$ trials per sample and control. (**D**) Relative TRAP activity in cell cultured medium of osteoclasts compared with M0 macrophages. $N = 3$ trials per sample and control. Data are presented as mean ± SEM. *** $p < 0.001$, compared with the hADMSCs group. M0: M0 macrophages; Oc: osteoclasts.
